# Normative data for human postural vertical: A systematic review and meta-analysis

**DOI:** 10.1371/journal.pone.0204122

**Published:** 2018-09-28

**Authors:** Laila B. Conceição, Jussara A. O. Baggio, Suleimy C. Mazin, Dylan J. Edwards, Taiza E. G. Santos

**Affiliations:** 1 Mae de Deus Hospital, Porto Alegre, Rio Grande do Sul, Brazil; 2 Department of Neuroscience and Behavioral Sciences, Ribeirao Preto Medical School, University of Sao Paulo, Ribeirao Preto, São Paulo, Brazil; 3 Federal University of Alagoas, Arapiraca, Alagoas, Brazil; 4 Department of Obstetrics and Gynecology, Ribeirao Preto Medical School, University of Sao Paulo, Ribeirao Preto, São Paulo, Brazil; 5 Moss Rehabilitation Research Institute, Elkins Park, Pennsylvania, United States of America; 6 School of Medical and Health Sciences, Edith Cowan University, Joondalup, Australia; University of Derby, UNITED KINGDOM

## Abstract

Perception of verticality is required for normal daily function, yet the typical human detection error range has not been well characterized. Vertical misperception has been correlated with poor postural control and functionality in patients after stroke and after vestibular disorders. Until now, all the published studies that assessed Subjective Postural Vertical (SPV) in the seated position used small groups to establish a reference value. However, this sample size does not represent the healthy population for comparison with conditions resulting in pathological vertical. Therefore, the primary objective was to conduct a systematic review with meta-analyses of Subjective Postural Vertical (SPV) data in seated position in healthy adults to establish the reference value with a representative sample. The secondary objective was to investigate the methodological characteristics of different assessment protocols of SPV described in the literature. A systematic literature search was conducted using Medline, EMBASE, and Cochrane libraries. Mean and standard deviation of SPV in frontal and sagittal planes were considered as effect size measures. Sixteen of 129 identified studies met eligibility criteria for our systematic review (n = 337 subjects in the frontal plane; n = 187 subjects in sagittal plane). The meta-analyses measure was estimated using the pooled mean as the estimator and its respective error. Mean reference values were 0.12°±1.49° for the frontal plane and 0.02°±1.82° for the sagittal plane. There was a small variability of the results and this systematic review resulted in representative values for SPV. The critical analysis of the studies and observed homogeneity in the sample suggests that the methodological differences used in the studies did not influence SPV assessment of directional bias in healthy subjects. These data can serve as a reference for clinical studies in disorders of verticality.

## Introduction

Spatial orientation is an important aspect of human function, and vertical alignment in relation to gravitational forces is considered the most common position for daily activities [1]. Maintenance of vertical posture is based on the integration of sensory and motor systems that provide information to the Central Nervous System (CNS) to construct and update an internal model of verticality [2,3].

The brain areas related to verticality perception described by neuroimaging studies involve the parietal cortex, superior and middle temporal gyrus, temporo-parietal junction, post central gyrus, inferior frontal gyrus, insula, and thalamus [4–11]. Altered verticality perception has been described in aged people [12], patients with vestibular disorders [13], Parkinson’s disease [14], idiopathic scoliosis [15], and stroke patients [5]. Recently, verticality perception was further associated with postural control and functionality in stroke patients [16].

Verticality perception can be assessed by three different modalities: subjective visual vertical (SVV), subjective postural vertical (SPV) and subjective haptic vertical (SHV). To assess SPV, the subjects remain seated on a tilting chair (eyes closed), and verbally instruct the examiner to set the chair to their perceived upright body orientation. This verticality perception is the primary modality correlated with postural control deficits in patients with CNS lesions [5,16].

Until now, there have been no reference values of SPV for a representative sample of the healthy adult population. Published studies in this area used small groups to establish reference values [17]. However, this sample size does not represent the healthy population. According to Lott et al. 1992 [18] and Reed et al. 1971 [19], a sample of at least 100 subjects, is required to estimate a reference values adequately. An alternative way to involve a greater sample is to analyze the data from the published studies using a systematic review with meta-analysis.

One consideration with between-study comparisons, is the variation in method for SPV evaluation. The principal methodological differences among studies are; the position of the volunteer during the SPV evaluation [5,20], type of equipment [21–23] and number of trials of the SPV test [5,21,23].

However, the impact of these variations on SPV result remains to be determined. Therefore, the primary objective of this study was to conduct a systematic review with meta-analysis of SPV data in seated healthy adults, to establish reference values with a representative sample. The secondary objective was to investigate the methodological protocol variants of SPV assessment, described in the literature.

## Material and methods

### Articles search and selection

The study followed PRISMA (Preferred Reporting Items for Systematic Reviews and Meta-Analyses) recommendations (S1 Checklist) [24]. Two researchers made a search of published articles from January 1980 to January 2018, in the Medline, EMBASE, and Cochrane databases independently from one another. The two researchers also analyzed the references of the studies. The keywords used for research were: “healthy subjects,” “vertical perception,” “verticality,” “postural vertical.”

The inclusion criteria were: randomized clinical trials, epidemiological studies (cross-sectional, cohort and case-control studies), and evaluation of the SPV in a seated position with no visual cues, and in healthy individuals. The articles that met the inclusion criteria were analyzed by two further reviewers, who entered into an agreement for the final inclusion of studies in this systematic review.

The data collected were: sample size, age, gender, method of SPV evaluation (type of chair; speed control; feet support; restriction of the volunteer at the chair; use of a neck brace; number of trials), values of the SPV in frontal and sagittal planes, and the country where the study was conducted.

To minimize the risk of bias, the methodological quality of the studies was described using the QUADAS (Quality Assessment of Diagnostic Accuracy Studies) tool. The QUADAS is a validated evidence-based tool for quality assessment, used in systematic reviews, to report the risk of bias and the study accuracy [25]. This tool contains 14 questions and 6 were selected based on the objectives of the present study. The selected questions of QUADAS were: (1) Was the spectrum of participants representative of the participants who will receive the test in practice? (2) Were selection criteria clearly described? (5) Did the whole sample or a random selection of the sample, receive verification using a reference standard of diagnosis, or, at least, confirmed verbally having no disease? (9) Was the execution of the reference standard described in sufficient detail to permit its replication? (12) Were the same clinical data available when test results were interpreted as would be available when the test is used in practice? (13) Were uninterpretable/intermediate test results reported?

### Meta-analysis

The meta-analyses for frontal and sagittal planes were performed following the considerations of Dodds et al. 2016 [26]. The mean and standard deviations of the SPV measures for frontal and sagittal planes were considered as effect size measures. A positive sign indicated clockwise SPV tilt in the frontal plane and forward SPV tilt in the sagittal plane; and a negative sign a counterclockwise SPV tilt in the frontal plane and backward tilt in the sagittal plane. Where necessary, the researchers contacted authors of relevant articles via electronic mail, requesting more information about the effect size measures.

Since the maximum likelihood calculation associated with the meta-analytic mean requires several studies to provide enough data to obtain accurate estimations, it would be recommended to use the source data [27]. Therefore, we calculated the pooled mean as estimator for the mean population, and its associated estimator correcting the bias among small number of observations (few studies observed in the literature) [26,28]. The estimation of the normality range considered the pooled mean +/- 2 standard deviations (Dataset and normative calculation in S1 Dataset). Heterogeneity among the included studies in each meta-analysis was tested with the Cochran Q test and the I^2^ statistical test. The I^2^ test quantifies the heterogeneity among studies, which can vary from 0% to 100% [29].

## Results

**Fig 1:**
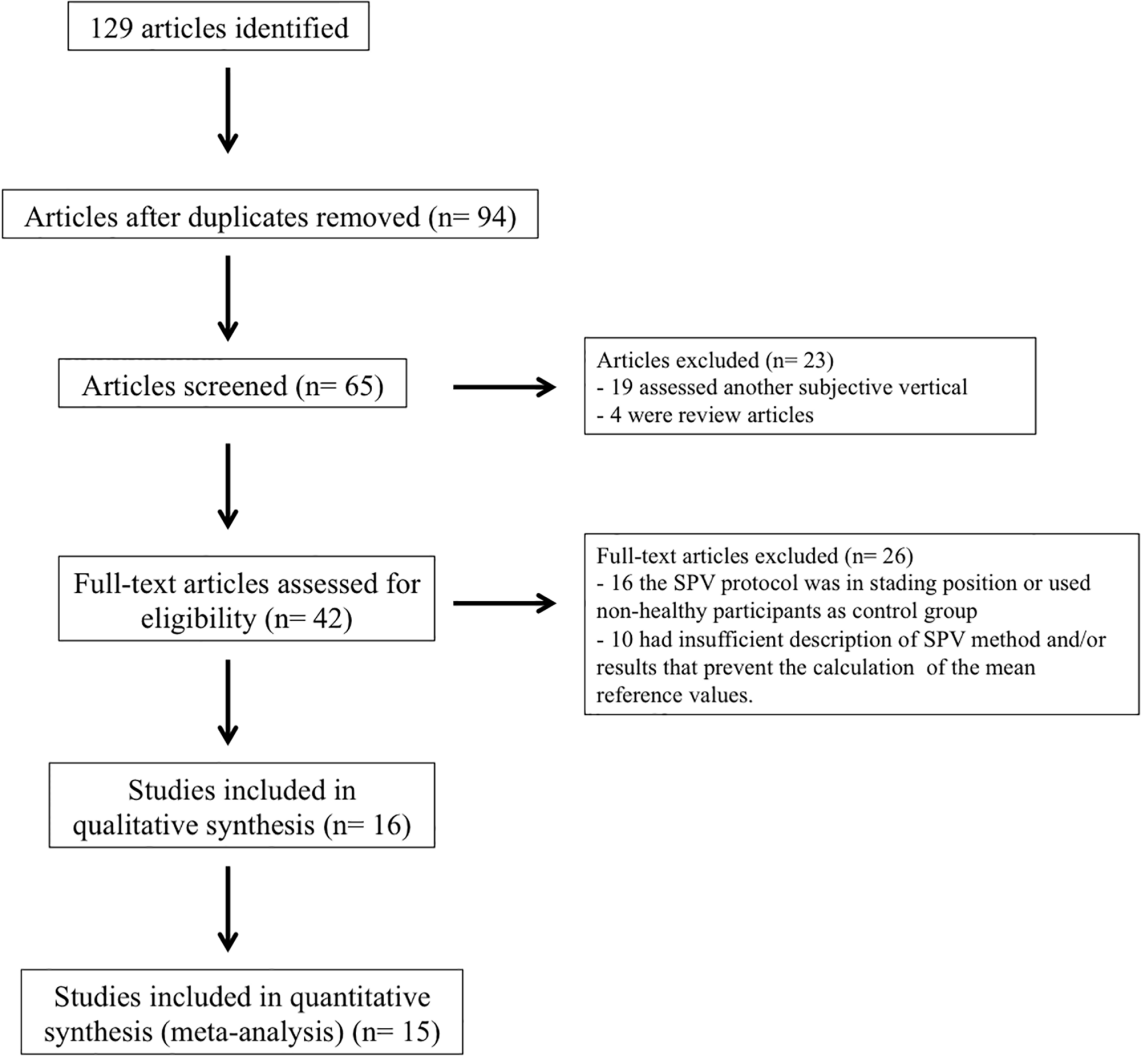
Flowchart from studies selection.

From the database searches, 89 articles were identified, and a further 40 studies were found through a search of references. After reading the titles and abstracts, the independent researchers selected 42 studies for the analysis of eligibility. From these, 26 were excluded for not meeting the inclusion criteria; 16 of which the SPV protocol was in standing position or used control groups with non-healthy participants; and 10 of which had insufficient description of SPV method and/or results that prevent the calculation of the mean reference values. Finally, 16 articles were included [5,12,13,21–23,30–39] which resulted in 434 subjects evaluated (Fig 1).

From these studies, three made the evaluation in frontal and sagittal planes [13,21,30] two in sagittal plane [12,34], and 11 in frontal plane [5,22,23,31–33,35–39]. Table 1 describes the data extracted from the articles.

**Table 1:** Characteristics of studies included in meta-analysis.

Author/ Year/ Reference	Country	n	Frontal SPV	Sagittal SPV	Type of chair	Who moves	Speed	Feet support	Restriction of volunteer	Number of trials	Age (years) Mean ±SD (range)	Gender	Visual absence
Mansfield et al., 2015 [22]	Canada	10	-0.33°±1.65°		Manual	Examiner	0.5°/s	Yes	Trunk and legs	6	65.3 (from 55 to 79)	4F, 6M	Darkness and blindfolded
Israël et al., 2012 [21]	France	10	-0.6°±4.2°	1.4°±4.2°	Motorized	Volunteer	45°/s (maximal speed)	Yes	3 belts (§)	48 (24 frontal; 24 sagittal)	^ (from 25 to 40)	^	Darkness
Barbieri et al., 2010 [12]	France	87		-0.76±1.22°	Manual	Examiner	1°-1.5°/s	Yes	Head, trunk, thighs and legs	10	^ (from 20 to 97)	38F, 49M	Blindfolded
Joassin et al., 2010 [36]	France	13	0.45°±1.02°		Manual	Examiner	1.5°-2°/s	Yes	Head and trunk	10	39.15±10.34	2F, 11M	Darkness
Saeys et al., 2010 [23]	Belgium	61	0.18°±1.55°		Motorized	Volunteer	^	No	Abdominal belt + side bar	4	49.77±22.52	35F, 26M	Blindfolded
Barbieri et al., 2008 [34]	France	12		0.78°±1.7°	Manual	Examiner	≈1.5°/s	Yes	Head, trunk, legs and feet	6	23.3±1.9	6F, 6M	Blindfolded
Pérennou et al., 2008 [5]	France	33	0.03°±0.9°		Manual	Examiner	≈1.5°/s	Yes	Head, trunk and legs	10	48,8±10,8	11F, 22M	Blindfolded
Mazibrada et al., 2008 [37]	England	20	-0.4°±0.8°		Manual	Examiner	1.5°/s^-1^	Yes	Head, shoulder, hips and legs	20	42±13	8F, 12M	Darkness and blindfolded
Aoki et al., 1999 [33]	England	22	-0.43°±1.5°		Motorized	Volunteer	^	Yes	Head, trunk and legs	4	43±15.6	11F, 11M	Darkness
Anastasopoulos et al., 1999 [31]	Greece	20	1.6°±1°		Motorized	Volunteer	2°-10°/s	Yes	Trunk	12 to 16	50.2±10.8	^	Eyes closed, method not specified
Pérennou et al.,1998 [38]	France	14	0.9°±0.3°		Manual	Volunteer	Self-regulated	No	No	^	54.7±3	5F, 9M	Darkness and blindfolded
Anastasopoulos et al.,1997 [32]	Germany/ Greece	20	-1.3°±1.4°		Motorized	Examiner /Volunteer	10°/s^2^	Yes	Trunk	12 to 16	50.2±10.8	^	Eyes closed, method not specified
Anastasopoulos et al., 1997 [30]	England	26	1°±1.7°	1.5°±2.2°	Motorized	Volunteer	1.5°/s	Yes	Head, trunk and legs	10	47.7±18	^	Eyes closed
Bisdorff et al.,1996 [35]	England/ Luxembourg	8	-0.4°±0.9°		Motorized	Volunteer	1.5°/s	Yes	Head and trunk	8	25.8±7.8	6F, 2M	Eyes closed
Bisdorff et al.,1996 [13]	England	52	0.12°±0.95°	0.16°±0.95°	Motorized	Volunteer	1.5°/s	Yes	Head and trunk	7 to 10	40.4 (from 21 to 80)	26F 26M	Eyes closed
Fukata et al., 2017 [39]	Japan	13 young 13 old	0.1±0.6 -0.1±1.1		Manual	Examiner	1.5°/s	No	Trunk	8	25,1± 2,3 (22–30) 67±5,1 (60–74)	7F; 6M 7F; 6M	Eyes closed

^(≈)^ approximately;

(^) unclear in the original article;

^(§)^ does not specify where; (F) female; (M) male.

Gender, educational level and age were described to influence verticality perception [12,40]. However, no study dichotomized the participants regarding educational level or gender of the subjects and few studies dichotomized the participants regarding age [12,39] preventing further analysis on the standardization of SPV under each subgroup with representative sample of subjects.

In all included studies, evaluations were made in the absence of visual input (Table 1). The majority of the studies stabilized the participants’ head and trunk but used different systems [5,12,13,21,22,30,33–37] and none used a neck brace. Another important aspect is the inclusion criteria of the healthy group. Only seven studies [12,21–23,34,35,37] described the inclusion criteria for the healthy group.

**Fig 2:**
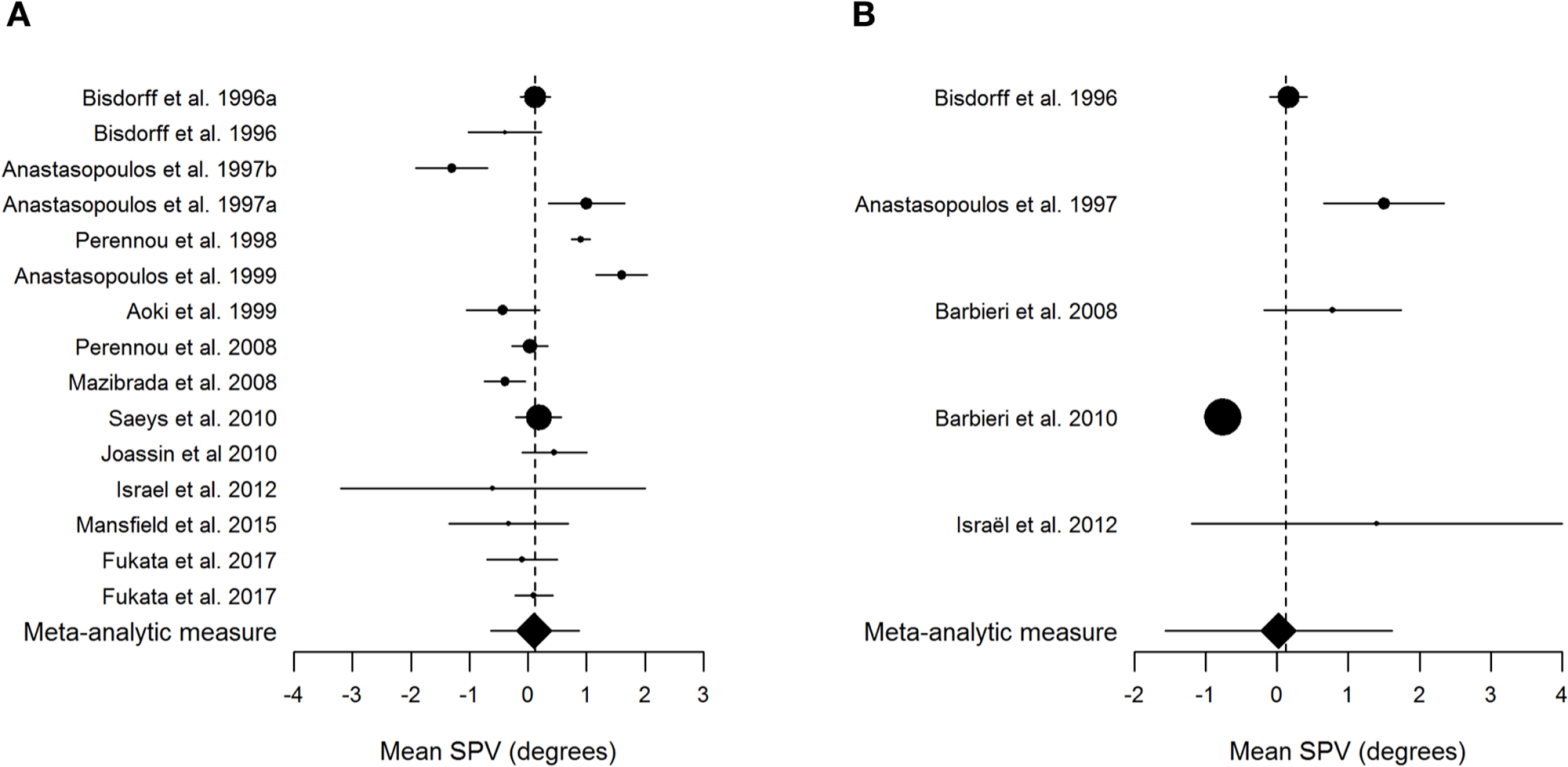
Forest plot from SPV values in frontal plane (left) and in sagittal plane (right). Black circles represent the mean and the horizontal bars extend from the lower limit to the upper limit of the 95% confidence interval of the mean. The size of the black circle corresponding to each study is proportional to the sample size. The estimated pooled mean is shown by the diamond.

**Fig 3:**
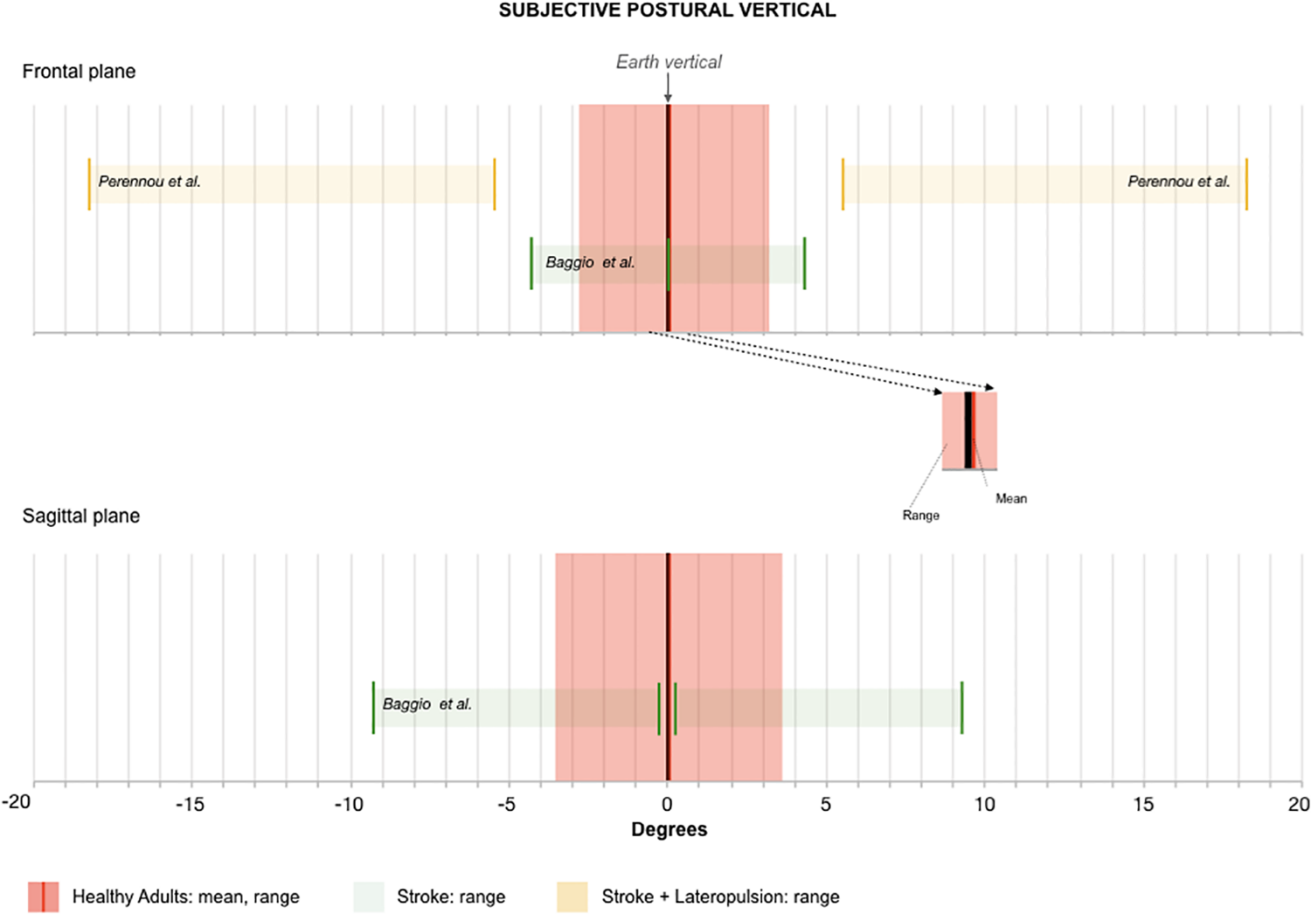
Illustration of the normative range (red) established by the present meta-analysis in the frontal plane (upper figure) and sagittal plane (lower panel). Additional data were included in the figure to illustrate previously published results of SPV in stroke patients (green). The minimum and maximal SPV for both sides (positive values: ipsilesional side; negative values: contralesional side) of stroke patients without lateropulsion described by Baggio et al. (2016) [16] show that the error range is vastly greater than normal, but that the minimum error range can fall within normal limits. The minimum and maximal SPV values of stroke patients with lateropulsion behavior described by Perennou et al. (2008) [5] in the frontal plane for both sides (positive values: ipsilesional side; negative values: contralesional side) are illustrated in yellow, and fall exclusively outside the normal range. These data support the high sensitivity of healthy human perception of postural vertical, and the disparity in neurological patients.

In Table 2, the evaluated items and corresponding results of QUADAS for the included studies are shown. The I^2^ value for frontal and sagittal planes were -0.77% and 13.95%, respectively. Negative values of I^2^ are considered equal to zero indicating no observed heterogeneity [29]. The reference value for the frontal plane was 0.12°±1.49° where the calculation of mean ± two standard deviations resulted in a range from -2.87° to 3.11°. The reference value for sagittal plane 0.02°±1.82° and the calculation of mean ± two standard deviations ranged from -3.61° to 3.66° (Table 3). The information of the effect size of each study, its respective 95% confidence interval, and the meta-analytic measure are presented in the Forest plot (Fig 2). There was no subgroup analysis regarding age or gender due to insufficient sample size to determine normative values. Fig 3 illustrates of the normative range established by the present meta-analysis in the frontal plane and sagittal plane. Additional data were included in the Fig 3 to illustrate previously published results of SPV in stroke patients with lateropulsion (a postural reactive lateral tilt related to verticality misperception) described by Pérennou et al. 2008 [5] and without lateropulsion described by Baggio et al. 2016 [16].

**Table 2:** Assessment of methodological quality of studies adapted from QUADAS tool.

Author/ Year	1	2	5	9	12	13
Mansfield et al., 2015 [22]	No	Yes	Yes	Yes	Yes	Yes
Israël et al., 2012 [21]	No	No	Unclear	Yes	Yes	Yes
Barbieri et al., 2010 [12]	No	Yes	Yes	Yes	Yes	Yes
Joassin et al., 2010 [36]	No	Yes	Unclear	Yes	Yes	Yes
Saeys et al., 2010 [23]	No	Yes	Yes	Yes	Yes	Yes
Barbieri et al., 2008 [34]	No	Yes	Yes	Yes	Yes	Yes
Pérennou et al., 2008 [5]	No	Yes	Unclear	Yes	Yes	Yes
Mazibrada 2008 [37]	No	Yes	Yes	Yes	Yes	Yes
Aoki et al., 1999 [33]	No	Yes	Unclear	Yes	Yes	Yes
Anastasopoulos et al.,1999 [31]	No	Yes	Unclear	Yes	Yes	Yes
Pérennou et al., 1998 [38]	No	Yes	Unclear	Yes	Yes	Yes
Anastasopoulos et al., 1997 [32]	No	Yes	Unclear	Yes	Yes	Yes
Anastasopoulos et al.,1997 [30]	No	Yes	Unclear	Yes	Yes	Yes
Bisdorff et al., 1996 [35]	No	Yes	Yes	Yes	Yes	Yes
Bisdorff et al., 1996 [13]	No	Yes	Unclear	Yes	Yes	Yes
Fukata et al., 2017 [39]	No	Yes	Yes	Yes	Yes	Yes

Questions of QUADAS tool: (1) Was the spectrum of participants representative of the participants who will receive the test in practice? (2) Were selection criteria clearly described? (5) Did the whole sample or a random selection of the sample, receive verification using a reference standard of diagnosis, or, at least, confirmed verbally having no disease? (9) Was the execution of the reference standard described in sufficient detail to permit its replication? (12) Were the same clinical data available when test results were interpreted as would be available when the test is used in practice? (13) Were uninterpretable/ intermediate test results reported?

**Table 3:** Statistical results from individual studies.

Frontal plane	n	Mean	SD	CI 95%		Weight
				IL	UL	
Mansfield et al. 2015 [22]	10	-0.33	1.65	-1.35	0.69	2.99
Israël et al. 2012 [21]	10	-0.60	4.20	-3.20	2.00	2.99
Joassin et al. 2010 [36]	13	0.45	1.02	-0.10	1.00	3.88
Saeys et al. 2010 [23]	61	0.18	1.55	-0.21	0.57	18.21
Pérennou et al. 2008 [5]	33	0.03	0.90	-0.28	0.34	9.85
Mazibrada et al. 2008 [37]	20	-0.40	0.80	-0.75	-0.05	5.97
Aoki et al. 1999 [33]	22	-0.43	1.50	-1.06	0.20	6.57
Anastasopoulos et al. 1999 [31]	20	1.60	1.00	1.16	2.04	5.97
Pérennou et al. 1998 [38]	14	0.90	0.3	0.74	1.06	4.18
Anastasopoulos et al. 1997 [32]	20	-1.30	1.40	-1.91	-0.69	5.97
Anastasopoulos et al. 1997 [30]	26	1.00	1.70	0.35	1.65	7.76
Bisdorff et al. 1996 [35]	8	-0.40	0.90	-1.02	0.22	2.39
Bisdorff et al. 1996 [13]	52	0.12	0.95	-0.14	0.38	15.52
Fukata et al. 2017 [39]	13	0.1	0.6	-0.23	0.43	3.88
Fukata et al. 2017 [39]	13	-0.1	1.1	-0.70	0.50	3.88
**Sagittal plane**						
Israël et al. 2012 [21]	10	1.40	4.20	-1.20	4.00	5.35
Barbieri et al. 2010 [12]	87	-0.76	1.22	-1.02	-0.50	46.52
Barbieri et al. 2008 [34]	12	0.78	1.70	-0.18	1.74	6.42
Anastasopoulos et al. 1997 [30]	26	1.50	2.20	0.65	2.35	13.90
Bisdorff et al. 1996 [13]	52	0.16	0.95	-0.10	0.42	27.81

(CI 95%) confidence interval 95%; (IL) inferior limit; (UL) upper limit; (SD) standard deviation.

## Discussion

This study represents the first systematic review of published SPV values in healthy adult individuals and provides useful reference data for the normative range for this perception. Prior studies have used the values the normality range described by Pérennou et al. 2008 [5], which describes the range of SVP in seated position from -2.5° to 2.5° in the frontal plane, defined as ‘mean ± two standard deviations ± measurement accuracy’. The authors stated that the measurement accuracy refers to the variability of the protocol. In the present work, the meta-analytic measure of 14 different studies in the frontal plane was 0.12°±1.49° (mean ± standard deviations) resulting in a range from -2.87° to 3.11°. Subjects outside the normative range would be thus considered non-normal, yet this does not necessarily denote clinically important.

The mean age of the healthy subjects investigated by Perennou et al. 2008 [5] (48.8±10.8 years) is comparable with the majority of the studies included in the present meta-analysis. Analyzing the available data in the literature, we could indicate the reference values that can be used in participants aged above 18 years old. It would be reasonable to additionally investigate the normative range of patients aged above 50 years separately to match the age of the sample observed in most disease-related studies. However, current literature does not provide sufficient studies of healthy aging subjects to perform this calculation, which highlights the need for future studies.

It is important to emphasize that risk of biased analysis must be included in the interpretation of all systematic reviews [41]. The strategies this study adopted to minimize the risk of methodological mistakes were following the PRISMA (Preferred Reporting Items for Systematic Reviews and Meta-Analyses) recommendations [24], and using the QUADAS tool [25,42]. There was a possibility of publication bias because of the propensity of published studies not being compatible with reality, as papers with homogeneous results can be preferentially published [43]. It is also possible that studies with high variability between healthy subjects or with small samples have been neglected for publication and, consequently, the low variability of result found may not be accurate. However, publication bias could not be measured.

Although the SPV is mostly assessed in seated position, it has been also evaluated in standing position. In this context, a small variability was also found in a study that evaluated the SPV in standing position in a sample of 60 healthy adults [20]. The authors found mean SPV values of 0.3°±1.0° in the sagittal plane and -0.2°±0.7° in the frontal plane and determined the normality range of SPV in standing position tilts from -1.7° to 2.3° in the sagittal plane, and from -1.6° to 1.2° in the frontal plane. Despite similar results, care is required when comparing the results of the SPV in standing and sitting positions. Some factors can interfere the assessment of SPV such as the possibility of movements from head and trunk in the standing position and the amount of somatosensory input provide in each evaluation.

All the studies included in this meta-analysis evaluated SPV in seated position but used one or more different methodological characteristics in the research design. Some of them used motorized chairs to do the test [13,21,23,30–33,35], and others used manual chairs [5,12,22,34,36–38]. The speed used to move the chair also varied within the sample. However, the majority of studies adopted a maximum displacement speed of 1.5°/s, which would help to eliminate semicircular canal stimulation, minimizing possible bias [5,12,13,30,34–36,39].

The number of times that each participant executed the SPV tests ranged from 4 to 24 trials in the investigated sample of studies, and these trials were divided equally in each direction of movement, both in frontal and sagittal planes. Future studies are necessary to determine the number of trials necessary to reliably assess SPV in healthy and patients with different neurological conditions.

Despite the different methodological aspects described above, homogeneity was observed within the postural vertical errors, suggesting that these methodological differences may not influence SPV assessment of directional bias, at least, in healthy subjects. We note that the normative values reported here result from comparable but slightly varying protocols, which accounts for the reported postural error variance in healthy subjects.

The methodological differences found among studies mostly refer to feet support and restrictions of trunk and legs. Regarding feet support, three studies did not use it to evaluate the SPV [23,38,39]. Since there is no study analyzing the influence of feet support on SPV it might be advisable not to use it. Among the included articles, the restriction was made varying the number and place of body fixations (Table 1). As a minimum, the restriction of the trunk and legs, as well as the maintenance of head alignment during the test is recommended to guarantee participants’ safety and to avoid postural reactions during the test. Although sensory inputs from trunk, legs and/or shoulders are important in the perception of verticality, even patients with paraplegia or tetraplegia present no directional bias in the orientation of postural vertical in the sitting position [1,36,44]. Moreover, this systematic review aimed at investigating the directional bias of postural vertical perception in the sitting position under a method of adjustment. The normative values of different types of analyses such as uncertainty degree of vertical perception [36,45], and different paradigms such as Aubert effect [1,31] or forced choice [46] should be also investigated in future studies.

Recently, the perception of visual vertical investigated in healthy individuals with and without the use of a neck brace [45] showed no differences between the compared conditions. Their findings reinforce the concept that these peculiarities in the evaluation are not capable of interfering the correct judgment of vertical perception directional bias in healthy subjects. Possible influences of methodological discrepancies are suggested to impact the perceptions of verticality after unilateral vestibular dysfunction [47] and encephalic lesions [17,48]. The correct functioning of the areas responsible for the integration of different sources of sensory input is needed to resolve possible conflicts using weighting of sensory information [49]. The relevance of absolute error (i.e. independence of direction from center/vertical) remains unclear. Calculation of mean error may be vastly different if one pays less attention to sign (+/-) of the error, but rather the magnitude; since the mean of two opposing errors can be zero. Furthermore, while we provide a reference range in this paper, we note a unilateral bias that may be relevant for stroke population according to the side of lesion.

Since SPV was shown to be a relevant perception for postural control [16,17,50], it is necessary to include SPV in the clinical evaluation of patients with postural imbalance. However, it is required to know the reference values for a healthy population to correctly diagnose alterations in this perception, and consequently, establish more effective rehabilitation strategies. We conclude that this systematic review and meta-analysis is an adequate reference for studies of postural vertical perception, and have provided the reference range within. This span of error is considered representative of ‘normal’ based on our meta-analysis”, and therefore may be used in future clinical studies as a normative reference.

## Supporting information

S1 Dataset Dataset and normative calculation. (XLSX)

S1 Checklist PRISMA checklist. (PDF)
